# Enhanced External Counterpulsation for Management of Postacute Sequelae of SARS-CoV-2 Associated Microvascular Angina and Fatigue: An Interventional Pilot Study

**DOI:** 10.1155/2023/6687803

**Published:** 2023-12-27

**Authors:** Eline Wu, Ali Mahdi, Jannike Nickander, Judith Bruchfeld, Linda Mellbin, Kristina Haugaa, Marcus Ståhlberg, Liyew Desta

**Affiliations:** ^1^Division of Cardiology, Department of Medicine Huddinge, Karolinska Institutet, Stockholm, Sweden; ^2^Heart and Vascular Center, Karolinska University Hospital, Stockholm, Sweden; ^3^Division of Cardiology, Department of Medicine Solna, Karolinska Institutet, Stockholm, Sweden; ^4^Department of Clinical Physiology, Karolinska Institutet, and Karolinska University Hospital, Stockholm, Sweden; ^5^Department of Infectious Diseases, Karolinska University Hospital, Stockholm, Sweden; ^6^Division of Infectious Diseases, Department of Medicine Solna, Karolinska Institutet, Stockholm, Sweden

## Abstract

**Background:**

Postacute sequelae of SARS-CoV-2 infection (PASC) are a novel clinical syndrome characterized in part by endothelial dysfunction. Enhanced external counterpulsation (EECP) produces pulsatile shear stress, which has been associated with improvements in systemic endothelial function.

**Objective:**

To explore the effects of EECP on symptom burden, physical capacity, mental health, and health-related quality of life (HRQoL) in patients with PASC-associated angina and microvascular dysfunction (MVD).

**Methods:**

An interventional pilot study was performed, including 10 patients (male = 5, mean age 50.3 years) recruited from a tertiary specialized PASC clinic. Patients with angina and MVD, defined as index of microcirculatory resistance (IMR) ≥25 and/or diagnosed through stress perfusion cardiac magnetic resonance imaging, were included. Patients underwent one modified EECP course (15 one-hour sessions over five weeks). Symptom burden, six-minute walk test, and validated generic self-reported instruments for measuring psychological distress and HRQoL were assessed before and one month after treatment.

**Results:**

At baseline, most commonly reported PASC symptoms were angina (100%), fatigue (80%), and dyspnea (80%). Other symptoms included palpitations (50%), concentration impairment (50%), muscle pain (30%), and brain fog (30%). Mean IMR was 63.6. After EECP, 6MWD increased (mean 29.5 m, median 21 m) and angina and fatigue improved. Mean depression scores showed reduced symptoms (−0.8). Mean HRQoL scores improved in seven out of eight subscales (+0.2 to 10.5).

**Conclusions:**

Patients with PASC-associated angina and evidence of MVD experienced subjective and objective benefits from EECP. The treatment was well-tolerated. These findings warrant controlled studies in a larger cohort.

## 1. Introduction

As individuals recover from COVID-19, some continue to report persisting symptoms weeks to months after acute COVID-19 referred to as postacute sequelae of SARS-CoV-2 (PASC) [1]. Recently, a consensus definition for PASC in adults was formulated as a condition which occurs in individuals with a history of probable or confirmed SARS-CoV-2 infection, three months from the onset, with symptoms that last for at least two months and cannot be explained by an alternative diagnosis [2, 3]. Common symptoms include, but are not limited to, fatigue, chest pain, palpitation, shortness of breath, sleep difficulties, anxiety/depression, and cognitive dysfunction. The symptom burden generally has an impact on everyday functioning [3–5]. Symptoms may be new onset following the initial recovery from an acute COVID-19 infection or persist from the initial illness. Moreover, symptoms could also fluctuate or relapse over time [2].

**Figure 1:**
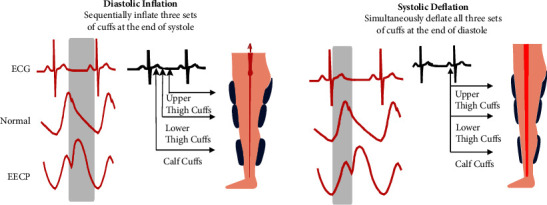
Schematic EECP showing sequential cuff inflation and deflation. Inflation and deflation are timed according to patient's R wave on ECG. Image used with permission from Vasomedical Inc.

Enhanced external counterpulsation (EECP) is a Food and Drug Administration-cleared, noninvasive outpatient therapy for patients with chronic stable angina and/or heart failure of ischemic etiology [6]. Standard therapy involves 35 one-hour sessions over seven weeks. Patients lie on a treatment table where compressive cuffs are securely wrapped around their lower extremities. These cuffs inflate in a distal-to-proximal sequence during diastole and deflate simultaneously just prior to the onset of systole (Figure 1) [7, 8]. The mechanical compressions of EECP induce physiologic and biochemical changes in the vasculature, leading to angiogenesis and increased coronary function [9, 10]. In patients with underlying cardiovascular disease, specifically refractory angina pectoris, EECP has been shown to significantly improve physical capacity, health-related quality of life (HRQoL), and six-minute walking distance (6MWD), all of which are independent markers associated with mortality benefits [11–13]. EECP also improves time to ST-segment depression, treadmill exercise duration, ejection fraction, and cardiac output/efficiency in this patient group [14]. Benefits of EECP are moreover shown in patients without underlying cardiovascular disease as a recent study showed improved exercise endurance and maximum oxygen uptake after three weeks of treatment in patients with chronic obstructive pulmonary disease [15]. Furthermore, EECP has also been known to enhance cerebral blood flow, collateralization in the ischemic regions of the brain, and cognitive function in ischemic stroke patients [16]. Mechanistically, EECP produces pulsatile shear stress, which has been associated with improvements in systemic endothelial function via boosting nitric oxide bioavailability and reducing proinflammatory mediators (e.g., tumor necrosis factor-alpha and monocyte chemoattractant protein-1) [9, 17]. Evidence is emerging that endothelial dysfunction is present in patients with PASC and may contribute to persisting symptoms [18]. Today, there is no evidence-based treatment targeting endothelial dysfunction in this patient cohort. To repurpose an available technology by testing the hypothesis that EECP reduces symptoms in patients with PASC that may be attributed to decreased endothelial function is reasonable. The aim of this study was to explore the effects of EECP on symptom burden, physical capacity, mental health, and HRQoL in patients with PASC-associated angina and microvascular dysfunction (MVD).

## 2. Methods

### 2.1. Study Design, Context, and Population

**Figure 2:**
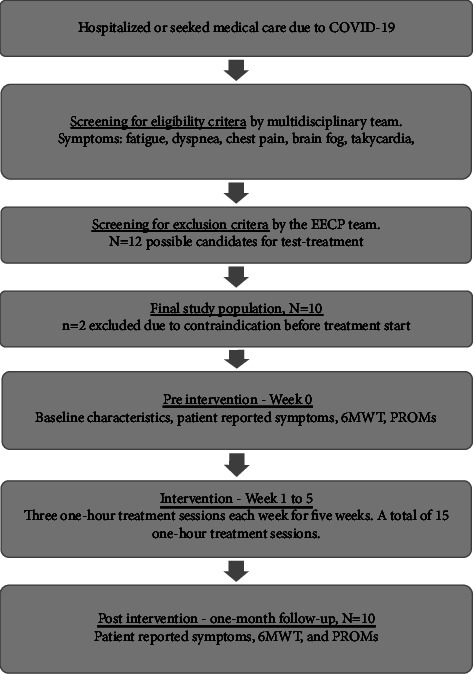
Flowchart of the recruitment of patients with postacute sequelae of SARS-CoV-2 and data collection. 6MWT, six-minute walk test; EECP, enhanced external counterpulsation; PROM, patient-reported outcome measure.

This is an interventional pilot study performed in 10 patients with PASC and MVD who underwent one modified course of EECP in a cardiology clinic at a tertiary university hospital. All patients were recruited after a diagnosis of PASC by a multidisciplinary team which was especially organized at the university hospital to optimize the management of this patient population. The multidisciplinary team identified patients who are suffering from PASC-associated angina and excluded patients who might have overlapping symptoms caused by other conditions (Figure 2). The study subjects underwent stress perfusion cardiac magnetic resonance imaging (CMRI) and/or invasive coronary angiography including measurement of microcirculatory resistance. The diagnosis of MVD was confirmed by stress perfusion CMRI and/or as index of microcirculatory resistance (IMR) ≥ 25. Two patients had signs of MVD via stress perfusion CMRI with borderline IMR and three patients underwent only CMRI due to young age with the intention of avoiding invasive assessment. All patients have evidence of MVD. Thereafter, patients were screened for exclusion criteria regarding EECP treatment according to local routine protocol. Exclusion criteria included advanced heart failure, severe valvular heart disease, severe peripheral arterial disease, ulcers on the lower extremities, bleeding disorders, severe pulmonary hypertension, or pregnancy [19, 20].

A total of 12 patients received study information and a test-treatment provided by a cardiac nurse before the final decision-making for study inclusion. Eventually, two patients were excluded due to pregnancy in one patient right before treatment start and a diagnosis of a painful schwannoma in the lower extremity of another patient. Consent was obtained from all patients. Ethical approval was given by the Swedish Ethical Review Authority (no. 2022-02001-01).

### 2.2. Intervention

A modified EECP treatment protocol (i.e., 15 one-hour sessions) with a Luminair EECP device (Vasomedical Inc., New York) with a maximum treatment pressure of 240 mmHg was given. One-hour treatment session was given three days per week over five weeks based on experiences from previous case reports [1, 21]. The treatment is noninvasive and involved electrocardiogram (ECG)-gated sequential leg compression with three sets of cuffs (like large blood pressure cuffs) wrapped around patient's lower extremities. Inflation and deflation are specifically timed to the R wave on the patient's electrocardiogram to optimize therapeutic benefit (Figure 1) [8]. The efficiency of counterpulsation is measured as the ratio between arterial diastolic-to-systolic pressures (i.e., D/S ratio) [8, 22]. EECP treatment was given during working days in an outpatient ward in the presence of a trained cardiac nurse. If needed, the cardiac nurse could at any time contact a cardiologist. The patients were monitored throughout the session with a single-lead ECG, blood pressure, heart rate, and oxygen saturation.

### 2.3. Clinical, Objective Data, and Patient-Reported Outcome Measurement

Baseline characteristics and sociodemographic data were collected by reviewing medical records and from clinical examination during the pre-EECP treatment visit. A 6-minute walk test (6MWT) was performed before and around one month after the completed EECP in accordance with recommendations by the American Thoracic Society [23]. Perceived exertion and pain or discomfort in the chest area, dyspnea, or tiredness in the legs was rated by the patient according to the Borg scales before and after the walk test. Heart rate and oxygen saturation were also measured [23]. Validated generic patient-reported outcome measures (PROM) were used. The hospital anxiety and depression scale (HADS) was used which contains 14 items with seven items forming an anxiety subscale and other items forming a depression subscale. The score ranges from 0 to 21 for each subscale, with lower scores indicating better outcomes [24]. RAND 36 was chosen to measure HRQoL, and it comprised 36 items that assess eight health concepts (i.e., physical functioning, role limitations caused by physical health problems, role limitations caused by emotional problems, social functioning, emotional well-being, energy/fatigue, pain, and general health perceptions). The score ranges from 0 to 100 for each subscale, with higher scores indicating better outcomes [25]. The PROMs were collected pre-EECP and at the one-month follow-up (Figure 2). The PROMs were in Swedish and distributed in paper format either through post or handed by the cardiac nurses.

### 2.4. Statistical Analysis

Descriptive statistics were mainly used to compute all data of interest. These data are shown as mean ± standard deviations or median and range as appropriate or in the case of categorical variables, as *n* (%). The paired *t*-test was used to analyze the 6MWD pre- and post-EECP. Wilcoxon signed rank test was used for nonparametric variables such as patient-reported PASC symptoms. The statistical significance level was set at *p* < 0.05. Statistical analysis was performed using IBM SPSS Statistics version 28.

## 3. Results

### 3.1. Patient Characteristics

Mean age of the patients was 50.3 years (min-max, 29–63 years) and women constituted 50% of the study participants. Traditional risk factors for cardiovascular disease were uncommon: hypertension (10%), hyperlipidemia (10%), and diabetes (0%) and four patients were former smokers (40%). Anxiety or depression was a pre-existing condition in 40% of the patients (Table 1). Most of the patients contracted COVID-19 during the first and the second waves of the pandemic. Only two were admitted to the hospital where they received intensive care (Table 2). The most commonly reported PASC symptoms were angina (100%), fatigue (80%), and dyspnea (80%). Other PASC symptoms include palpitation (50%), concentration impairment (50%), muscle pain (30%), and brain fog (30%). Sleeping and visual disturbance were rare (10%). Around 70% of the patients were suffering from four or more types of symptoms. At baseline, the average IMR was 63.6 (ref < 25) and many had a moderate to severe MVD according to invasive physiologic assessment (Table 2).

### 3.2. Patient-Specific Diastolic/Systolic Ratio Trends

**Figure 3:**
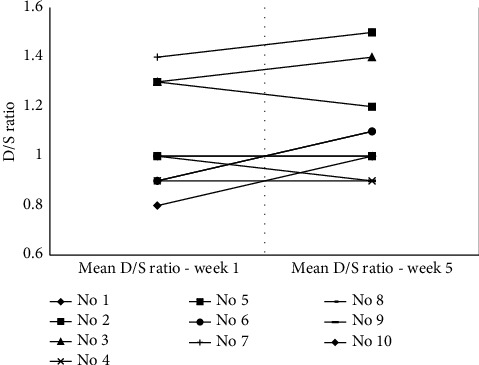
Individual patterns of the mean arterial diastolic-to-systolic pressures (D/S ratios) in the first and last weeks of the treatment period (*N* = 10).

The readings of vital parameters, i.e., blood pressure, heart rate, and oxygen saturation, were stable during the sessions. The mean D/S ratio in the first week was 1.06 and 1.10 in the fifth week. At the individual level, the mean D/S ratio improved over time in five patients; meanwhile, it was unchanged in three patients and decreased in two patients (Figure 3).

### 3.3. Changes in Symptom Burden

**Figure 4:**
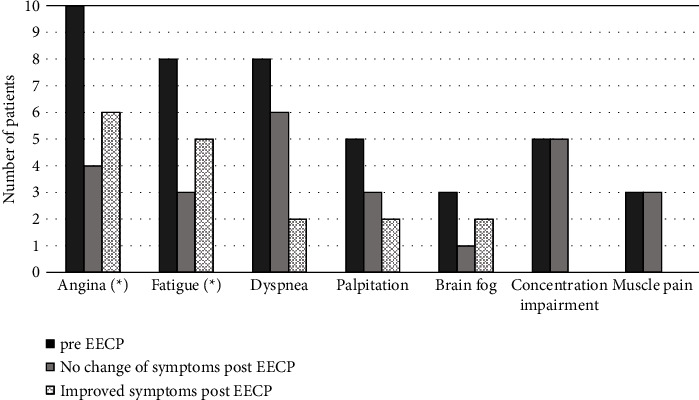
Patient-reported postacute sequelae of SARS-CoV-2 symptoms at baseline and at completion of modified enhanced external counterpulsation treatment (*N* = 10). Significant improvement in both angina and fatigue among the patients. ^*∗*^*p* < 0.05.

The most pronounced improvements after EECP were significant reduction in reported angina symptoms (*p* < 0.05) and reported fatigue (*p* < 0.05) (Figure 4). Improvement was also reported in dyspnea, brain fog, and palpitations. However, no change was seen regarding muscle pain and concentration impairment in those who suffered from it at baseline. One patient completely recovered and reported no symptoms following EECP. None of the patients experienced worsening of symptoms after EECP. Overall, the burden of PASC symptoms per patient was significantly reduced (*p* < 0.05). An improvement in working capacity was also observed whereby two patients went back to work part-time from a full-time sick leave at baseline.

### 3.4. Physical Capacity

Baseline mean 6MWD was 498.5 m and after intervention 528 m. Patients improved their walking distance after EECP (mean 29.5 and median 21 m) (Table 3). However, the improvement was not statistically significant. They were able to perform the 6MWT post-EECP on average with less exertion (15 vs 14.5) and less angina/dyspnea (3 vs 2.5) according to the Borg scales. Heart rate and oxygen saturation were stable after the walk test, both during pre- and post-EECP 6MWTs.

### 3.5. Psychological Distress and Health-Related Quality of Life

The level of depression was reduced after treatment with a mean score of −0.8 (Table 3). In contrast, the level of anxiety showed no improvement. Mean HRQoL scores improved in seven out of eight subscales (+0.2 to 10.5). The largest improvement was seen in the subscales of vitality (+10.5), social function (+8.6), and physical function (+7.5). No improvement was seen in the subscale of the emotional role functioning since seven patients reported no change and three experienced a deterioration compared to baseline.

## 4. Discussion

This pilot study demonstrates that patients with PASC-associated angina and MVD experienced subjective and objective benefits from EECP. The treatment was well-tolerated in all patients who completed the protocol successfully.

The PASC symptoms which improved significantly after the treatment were angina and fatigue. Coronary MVD was present in all patients and microvascular angina seems to be the clinical manifestation of myocardial ischaemia in patients with PASC. Previous reports have showed that a substantial proportion of patients treated in COVID-19 units suffer from chest pain/angina which might be due to MVD as demonstrated in our study [26, 27]. Since standard EECP treatment provides positive effects through mechanisms such as improvement of endothelial function and coronary perfusion and flow in patients with refractory angina pectoris, it could offer considerable benefits by alleviating symptoms to these patients as well. Growing evidence suggests that improvement in endothelial function represents an important mechanism for the clinical benefit observed with EECP. Central to this concept is the increase in organ blood flow observed during treatment [10, 28]. EECP-induced increases in blood flow translate into enhanced endothelial shear stress. Increased shear stress represents a major stimulus for endothelial nitric oxide release [29]. Indirect evidence for the existence of a beneficial effect of EECP on endothelial function stems from the observation that the level of diastolic augmentation, or the effectiveness ratio (D/S ratio), tends to increase over the course of treatment. Of concern, might be if the modified protocol of EECP is efficient enough to have sustainable effects in these patients as it is significantly shorter compared to the standard EECP protocol used in patients with refractory angina pectoris with evident potential long-term benefit [13, 30].

A considerable proportion of patients experienced less fatigue after EECP treatment which is also demonstrated by the findings of RAND-36 vitality scale which is a general measure of energy/fatigue. Social functioning also improved which implies that the patients were able to maintain general social interaction in and outside home after EECP. Post-COVID-19 fatigue is defined as a decrease in physical and/or mental performance resulting from changes in central, psychological, and/or peripheral factors resulting from COVID-19 disease [31]. Since patients with fatigue may also suffer from brain fog, myalgia, and depression/anxiety, more research is needed to identify treatment options which might be helpful against these symptoms and conditions [32]. Case reports and smaller studies have described EECP as helpful for neurocognitive symptoms in patients with PASC-associated symptoms [1, 21, 33, 34].

Improvement in 6MWD and all physical subscales of RAND 36 (e.g., physical function, physical role functioning, bodily pain, and general health) after a modified EECP treatment might suggest that this treatment could be a potentially promising intervention for this group of patients which currently lack specific disease modifying evidence-based therapy options. A recent study indicated that physical exercising will play a substantial role in the rehabilitation of COVID-19 survivors suffering from PASC as it positively affects both physical and mental dimensions as well as work ability [35]. Another study reported that EECP improved the quality of life and fatigue in patients with PASC and allowing them to resume normal daily activities and return to work [34], which are findings similar to ours. In the present study, the physical limitation patients had hindered them from exercising to the extent they needed to gain benefits. As EECP achieves several physiological changes through a passive means similar to those from physical training, it most likely triggers a favorable process that would assist patients perform regular physical exercise. During EECP, the patient's blood pressure and heart rate are maintained at the resting level. Since the treatment is a form of passive exercise, the sympathetic nervous system is not stimulated, which could be a potential way to trigger a favorable process supporting patients to consider and start exercising since the initial ability to be physical active is low.

Our study showed a slightly positive change in depression scale after EECP. However, moderate levels of both anxiety and depression in general were also measured, both at baseline and after intervention, among the patients, which is a worrying sign. Even though reports showed that survivors of COVID-19 are at risk of psychiatric sequelae (e.g., anxiety, depression, posttraumatic stress disorder, cognitive deficits, and sleep disturbances), these symptoms generally improve over time [36, 37]. Even so, to re-evaluate the mental state of patients with PASC could be necessary and using validated screening tools could be preferable as they provide cut-off values with indications of whether the need for psychological support exists or not.

### 4.1. Limitations

This study has a few limitations that need to be considered. Limitations include the small sample size, the lack of a control group, and randomization. Moreover, endothelial function or change in microvascular function was not assessed as a part of the study. Future research should include a control group and assessment of endothelial function before and after treatment. In addition, we are not sure if the modified EECP protocol we used in this pilot study is an optimal protocol for this patient group as it is significantly shorter and less intensive compared to the standard protocol (15 sessions vs 35 sessions) used in patients with refractory angina pectoris. This protocol was chosen due to its use in previous published case reports [1, 21].

## 5. Conclusions

EECP treatment was associated with a reduction in symptom burden, improvement of physical capacity, and HRQoL in patients with PASC-associated angina and evidence of MVD. These findings suggest that EECP may be beneficial for the management of PASC. Investigating EECP treatment in controlled studies with larger cohorts of patients with PASC is highly warranted.

## Figures and Tables

**Table 1:** Sociodemographic and clinical data at baseline in patients with postacute sequelae of SARS-CoV-2 (*N* = 10).

	*n* (%), unless otherwise stated
*Sociodemographic data*	
Sex	
Men	5 (50)
Women	5 (50)
Age	
Mean (min-max)	50.3 (29–63)
Marital status	
Single/widow/divorced	3 (30)
Married/partner, not living together	7 (70)
*Cardiovascular risk factors*	
Smoking habit	
Nonsmoker	6 (60)
Ex-smoker	4 (40)
Body mass index (kg/m^2^)	
≥25	9 (90)
Exact value, mean (Sd)	28.6 (3.6)
Diabetes mellitus	0
Hypertension	1 (10)
Hyperlipidemia	1 (10)
Myocardial infarction	0
*Other co-morbidities*	
Fibromyalgia syndrome	1 (10)
Polyarthritis	1 (10)
Polymyalgia rheumatic	0
Prior anxiety/depression	4 (40)
Pulmonary disease	0
*Clinical data*	
Systolic blood pressure (mmHg)	
Mean (Sd)	127 (8.1)
Diastolic blood pressure (mmHg)	
Mean, (Sd)	78 (6.8)
Heart rate (bpm)	
Mean (Sd)	69 (13.7)
Ejection fraction	
≥50%	10 (100)
NT-proBNP (ng/L)	
Mean (Sd)	71.5 (50.81)
*Pharmacologic treatments*	
Angiotensin-converting-enzyme-inhibitors	1 (10)
Angiotensin receptor blockers	1 (10)
Anticoagulants	0
Antidepressant	3 (30)
Antiplatelet agents	1 (10)
Asthma inhalers	
Short-acting	6 (40)
Long-acting	0
Both	0
Beta blockers	5 (50)
Calcium channel blockers	6 (60)
If-inhibitor	1 (10)
Lipid-lowering agents	3 (30)
Long-acting nitrates	3 (30)

NT-proBNP, N-terminal of the prohormone brain natriuretic peptide.

**Table 2:** Baseline characteristics related to postacute sequelae of SARS-CoV-2 (*N* = 10).

	*n* (%), unless otherwise stated
Contract COVID-19 infection	
First wave	5 (50)
Second wave	4 (40)
Third wave	1 (10)
Admission to hospital	2 (20)
Microvascular dysfunction (MVD)	10 (100)
Verification of MVD through	
Stress perfusion CMRI	10 (100)
IMR	7 (70)
IMR measurement	
Mean (Sd, min-max)	63.6 (62.9, 21–184)
Grading of IMR	
Normal, <25	2 (28.5)
Mild, 25–30	0
Moderate, 31–50	3 (43.0)
Severe, 51 or more	2 (28.5)
No data	3

CMRI, cardiac magnetic resonance imaging; IMR, index of microcirculatory resistance; MVD, microvascular dysfunction.

**Table 3:** Change from baseline in walking distance and patient-reported outcome measures in patients with postacute sequelae of SARS-CoV-2 after a modified enhanced external counterpulsation treatment.

Patient	Sex	Physical capacity, 6MWT	Psychological distress, HADS, two subscales		Health-related quality of life, RAND-36, eight subscales							
		WD meters	Anxiety score	Depression score	PF score	RP score	BP score	GH score	VT score	SF score	RE score	MH score
1	F	−2.5	−4	−1	10	0	20	−19	−10	12	0	4
2	F	35	−3	0	0	0	0	0	10	37	0	0
3	M	9	0	1	−2	0	10	−15	5	0	0	0
4	F	16	0	1	0	25	−10	−5	5	0	−67	−12
5	F	−38	6	2	5	0	−33	10	−10	0	−100	−16
6	M	33	2	−1	−5	0	13	−1	5	0	0	0
7	M	19	4	−3	0	−50	2	0	30	0	0	12
8	M	23	−6	−6	35	100	10	30	45	25	0	20
9	F	27	0	−1	0	0	−23	−3	−15	12	−100	0
10	M	173.5	2	0	20	0	13	8	40	0	0	20
N = 10												
Mean (SD)		29.5 (54.9)	0.1 (3.7)	−0.8 (2.3)	6.3 (12.4)	7.5 (37.4)	0.2 (17.2)	0.5 (13.7)	10.5 (21.1)	8.6 (13.1)	−26.7 (43.9)	2.8 (11.9)

6MWT, six-minute walk test; BP, bodily pain; F, female; GH, general health; HADS, hospital anxiety and depression scale; M, man; MH, mental health; PH, physical health; RE, role disability due to emotional health; RP, role disability due to physical health; SD, standard deviation; SF, social function; VT, vitality; WD, walking distance.

## Data Availability

The data that support the findings of this study are available from the corresponding author upon reasonable request.
